# Bioactivity characterization of *Lactobacillus* strains isolated from dairy products

**DOI:** 10.1002/mbo3.280

**Published:** 2015-07-27

**Authors:** Babak Haghshenas, Yousef Nami, Minoo Haghshenas, Norhafizah Abdullah, Rozita Rosli, Dayang Radiah, Ahmad Yari Khosroushahi

**Affiliations:** 1 Institute of Bioscience, University Putra Malaysia43400 UPM Serdang, Selangor, Malaysia; 2 School of Medicine, Shahid Beheshti University of Medical SciencesTehran, Iran; 3 Chemical and Environmental Engineering Department, Faculty of Engineering, University Putra Malaysia43400 UPM Serdang, Selangor, Malaysia; 4 Drug Applied Research Center, Tabriz University of Medical SciencesTabriz, Iran; 5 Department of Pharmacognosy, Faculty of Pharmacy, Tabriz University of Medical SciencesTabriz, Iran

**Keywords:** Colostrum, *Lactobacillus*, molecular method, probiotic

## Abstract

This study aimed to find candidate strains of *Lactobacillus* isolated from sheep dairy products (yogurt and ewe colostrum) with probiotic and anticancer activity. A total of 100 samples were randomly collected from yogurt and colostrum and 125 lactic acid bacteria were isolated. Of these, 17 *Lactobacillus* strains belonging to five species (*L. delbrueckii*, *L. plantarum*, *L. rhamnosus*, *L. paracasei*, and *L. casei*) were identified. *L. plantarum* 17C and 13C, which isolated from colostrums, demonstrated remarkable results such as resistant to low pH and high concentrations of bile salts, susceptible to some antibiotics and good antimicrobial activity that candidate them as potential probiotics. Seven strains (1C, 5C, 12C, 13C, 17C, 7M, and 40M), the most resistant to simulated digestion, were further investigated to evaluate their capability to adhere to human intestinal Caco-2 cells. *L. plantarum* 17C was the most adherent strain. The bioactivity assessment of *L. plantarum* 17C showed anticancer effects via the induction of apoptosis on HT-29 human cancer cells and negligible side effects on one human epithelial normal cell line (FHs 74). The metabolites produced by this strain can be used as alternative pharmaceutical compounds with promising therapeutic indices because they are not cytotoxic to normal mammalian cells.

## Introduction

Colostrum is defined as the first milk is secreted by the mammary gland in the initial 24–96 h of the postpartum period (Solomons 2002). The production varies depending on the animal species. This initial secreted milk is a rich source of nutrients (protein, vitamins, fat, lactose and minerals), antimicrobial substances and growth factors. It also contains a high diversity of probiotic bacteria such as *Lactobacillus* and *Bifidobacterium* strains (Soccol et al. 2010; De Dea Lindner et al. 2011), which have been used widely in functional or probiotic foods.

The majority of probiotic bacteria belong to the lactic acid bacteria (LAB) group. LAB exhibit fermentation activities and have been used in food preservation for thousands of years. The species of *Lactobacillus* genus are the most famous group of LAB that are recognized as probiotics (Haghshenas et al. 2014; Nami et al. 2014a). *Lactobacillus* strains exhibit health-promoting and preservation activities (Dubernet et al. 2002) and are used as a starter culture to enhance the texture, flavor, and nutrition value of some products, such as cheese, sourdough, wine, beer, silage, fermented plant, and meat (Singh et al. 2009; Giraffa et al. 2010). Specific probiotic strains have positive effects on atopic eczema (Bunselmeyer and Buddendick 2010), irritable bowel syndrome (Parkes 2010), diarrhea (Binns and Lee 2010), antibiotic-related diarrhea (Friedman 2012), vaginal infections (Reid and Bocking 2003), inflammatory bowel disease (Geier et al. 2007), and cancers (Nami et al. 2014b,c; Serban 2014; Haghshenas et al. 2015) by stimulating the immune mechanisms and balancing the human microbiota composition. Moreover, certain strains significantly affect the bio-availability of such nutrients as magnesium and calcium in the human body (Young and Huffman 2003).

Probiotic bacteria are resistant to gastrointestinal conditions (low pH and high concentrations of bile salts) (Sahadeva et al. 2011). Some LAB carry antibiotic resistance genes and thus exhibit high resistance against antibiotics (Temmerman et al. 2003).

For probiotics to be capable of inducing to promote effects on host health, they must tolerate environments with high concentrations of bile salts and low pH and display high antimicrobial activities (Biradar et al. 2005). Therefore, this study aimed to screen the yogurt and ewe colostrum microbiota to find new strains of the *Lactobacillus* genus with high probiotic capability and anticancer activity by employing suitable and effective biochemical and morphological assays.

## Materials and Method

### Sampling, isolation, and morphological/biochemical characterization

A total of 100 samples from traditional yogurt and ewe colostrum were used for the isolation of the probiotic bacteria. Colostrum samples were obtained from the first milking of the postpartum and the starter culture used for making yogurt was from sheep's traditional yogurt. For isolation of bacteria, 1 mL of each dairy sample was suspended in 2% w/v sodium citrate solution and homogenized by using a Stomacher 400 Circulator (2 min) (Seward, Inc., London, England). Then, 1 mL of the samples was added to 10 mL of MRS (de Man, Rogosa and Sharpe) broth (Difco Laboratories, Detroit, Mich., USA) as the specific growth medium for *Lactobacillus* strains. After 24 h of anaerobic growth (37°C), 0.02 mL of the diluted solutions was spread for 48 h on MRS agar media.

Colonies obtained on MRS agar were extracted and transferred to 10 mL of broth culture for the enrichment step. After 24 h of incubation (37°C), screening for isolates was performed through a primary morphological test with the use of a fluorescent microscope (BX61; Olympus) (Olympus, Tokyo, Japan) and biochemical tests, such as Gram staining and catalase tests. The isolates were stored in 30% (w/v) glycerol and 10% (w/v) skim milk at −70°C (Mirzaei and Barzgari 2012).

### Amplification of 16S rRNA

The amplification of 16S rRNA of *Lactobacillus* strains was performed by using one primer pair (16F27 5′-AGAGTTTGATCMTGGCTCAG-3′ and 16R 5′-TACCTTGTTAGGACTTCACC-3′), as previously reported by Mirzaei and Barzgari (2012). These primers are genus-specific and can directly amplify the 16S rRNA (1500 bp) of *Lactobacillus* strains. The amplification was performed with a 25 *μ*L final volume containing 0.4 *μ*mol/L primer, 40 ng of chromosomal DNA, and master mix (Ampliqon, Herlev, Denmark). The PCR program cycles were set up as follows: denaturation at 95°C for 4 min, 32 cycles of 94°C for 1 min, 58°C for 1 min, 72°C for 95 sec, and a final extension at 72°C for 5 min.

### Sequencing of amplified 16S rRNA

The amplified 16S rRNA were purified by using a QIAquick PCR purification kit (Qiagen, Hilden, Germany) according to the manufacturer's instructions and then sequenced by a Korean sequencing company (Macrogene). The isolates were then identified and discriminated by blasting their sequences with BLAST software (http://blast.ncbi.nlm.nih.gov/Blast.cgi). The strains were then compared with the sequences deposited in NCBI and GenBank.

### Low pH and high concentrations of bile salts tolerance tests

Bacterial stocks were incubated in 5 mL of MRS growth medium at 37°C for 24 h. Then, 200 *μ*L of bacterial media were incubated in 5 mL of fresh medium for another 24 h. Bacterial cells were suspended by centrifuging at 2000*g* for 15 min and then washed twice with PBS (Phosphate-buffered saline). Thereafter, the cells were re-suspended in adjusted PBS (1 mL) with pH 2.5 or media-THIO (thioglycollate media) broth (37°C) for 3 h. The media-THIO broth was prepared by mixing MRS broth with 0.3% (w/v) oxgall and 0.2% (w/v) sodium thioglycollate (Sigma, St. Louis, MO, USA). The resistant cells were isolated, and their survival rates were calculated by using the pour plate technique in agar media; the obtained rates were compared with those of strains incubated in normal PBS for 0 and 3 h (Walker and Gilliland 1993).

In the pour plate method, the isolates were incubated in MRS broth medium at 37°C for 48 h after undergoing maximum serial dilution. The single colonies were counted, and their survival rates were measured (Charteris et al. 1998). In this method, the mean data were calculated by conducting the experiment twice with three iterations for each run. The mean values were considered for each bacterial isolate.

### Antimicrobial activity assay

A modified agar diffusion method described by Bauer et al. (1966) was used to determine antimicrobial activity. After overnight incubation in MRS broth medium at 37°C, the isolates were centrifuged for 10 min at 14,000*g*. The supernatants were filtered by using a 0.2 *μ*m filter. Then, 50 *μ*L of filtered of neutralized supernatant were added to 7 mm diameter wells created on a MRS agar plate preinoculated with indicator pathogens. The agar was incubated at 37°C overnight. The clear zone formation indicated a positive antimicrobial activity of isolated metabolites on the pathogens (Bauer et al. 1966). This experiment was performed against some clinically important human pathogens including *Salmonella typhimurium* (14028), *Staphylococcus aureus* (ATCC 25923), *Escherichia coli* (026), *Bacillus cereus* (ATCC 11778), *Listeria monocytogenes* (PTCC 1163), *Klebsiella pneumoniae* (ATCC 10031), and *Shigella flexneri* (PTCC 1234).

### Antibiotic susceptibility assay

The disk diffusion method was used to determine bacterial susceptibility against clinically important antibiotics including chloramphenicol (30 *μ*g), tetracycline (30 *μ*g), erythromycin (15 *μ*g), ampicillin (10 *μ*g), gentamycin (10 *μ*g), clindamycin (2 *μ*g), sulfamethoxazol (15 *μ*g), penicillin (10 *μ*g), and vancomycin (30 *μ*g). The isolates were completely incubated in Mueller-Hinton agar plates, and the antibiotic disks were placed on plates with the use of sterilized forceps and then incubated at 37°C for 18–24 h. Diameters of clear zones around disks were measured by using a digital caliper. According to the results and disk producer guidelines (NCCLS, 2007), the isolates were classified into sensitive, intermediate, and resistant groups.

### Survival in simulated in vitro digestion

To assess in vitro digestion, the method previously described by Seiquer et al. (2001) was used with some modifications. To recreate the gastric digestion, pepsin with a final concentration of 5% (w/v) was added to the samples, the pH values of which were adjusted to 3.0. The samples were incubated for 120 min at 37°C with gentle agitation at 110 rpm. To simulate intestinal digestion, the samples were adjusted to pH 6.0, and solutions of bile salts and pancreatin were added at final concentrations of 0.3% and 0.1% (w/v), respectively. The samples were incubated at 37°C for 180 min with gentle agitation at 110 rpm. To determine cell count, the samples were removed before and after gastric and intestinal digestion, and the aliquots were serially diluted and plated in triplicate on MRS agar. The plates were incubated for 48 h under anaerobic conditions.

### Adhesion of LAB to Caco-2 cells

Bacteria were evaluated for their adhesion capability to the human colon carcinoma cell line Caco-2. The cells were cultured in RPMI medium supplemented with 10% heat-inactivated fetal bovine serum and 1% penicillin–streptomycin mixture. Cells were cultured on 24-well tissue culture plates and incubated at 37°C in 5% CO_2_ under a relatively humidified atmosphere until a confluent monolayer was formed.

Before the adhesion assay, the media in the wells containing a Caco-2 cell monolayer were removed and replaced once with fresh antibiotic-free RPMI. Thereafter, 1 × 10^7^ cfu/mL of bacteria was added to each well with a total volume of 1 mL and then incubated for 3 h at 37°C under an atmosphere of 5% (v/v) CO_2_. To remove nonattached bacterial cells, the wells were washed thrice with a sterile prewarmed PBS solution. To detach the bacteria cells from the wells, 1 mL of 1% (v/v) Triton X-100 was added to each well, and the mixture was stirred for 10 min. To measure the viable cell count, the cell suspension was plated onto MRS agar and incubated at 37°C under anaerobic conditions. This assay was performed in triplicate.

### MTT assay

The cytotoxicity of the isolated *Lactobacillus* strains to tumor/normal cells was evaluated by using the microculture tetrazolium [MTT, 3-(4,5-dimethylthiazol-2-yl)-2,5-diphenyltetrazolium bromide] assay. Briefly, HT-29 and FHs 74 cells (1.2 × 10^4^ cell/well) were seeded into each well of a 96-well microplate with RPMI growth medium. After reaching 50% confluence, 24 h after seeding, the cells were treated with the filtered supernatant of the isolated strain at a time point of 24 h. After treatment, the medium was replaced with 200 *μ*L of fresh medium containing 50 *μ*L of MTT solution (2 mg/mL in PBS) and then incubated for an additional 4 h at 37°C. After incubation, the MTT mixture was carefully removed, and 200 *μ*L of dimethyl sulfoxide and 25 *μ*L of Sorenson's glycine buffer (0.1 mol/L glycine, 0.1 mol/L NaCl at pH 10.5) were added to each well. The mixture was then incubated for 30 min. Finally, the absorbance of each well was measured after 30 sec of shaking by using a microplate reader (ELx 800; Biotek, Winooski, VT, USA) at 570 nm. The cells were treated with MRS (bacterial culture medium) and Taxol (Tocris Cookson Ltd., Bristol, UK) (anticancer drug as a reference) as negative and positive controls, respectively.

### Apoptotic cell detection by fluorescent microscopy

To detect apoptotic cells, sterile cover slip slides were placed into each well of a six-well culture plate. Then, 2 mL of HeLa cancer cells (1.2 × 10^4^ cells/mL) were added to each well and then incubated at 37°C under 5% CO_2_. After reaching 50–60% confluence, 500 *μ*L of the cell-free filtered supernatant of selected strains were added to each well and incubated for 24 h at same condition. The treated cells were washed with prewarmed tissue culture media 24 h after seeding and were carefully removed and replaced with freshly prepared fixative solution (prewarmed RPMI containing 4% formaldehyde).After the plates were incubated for 5 min at 37°C, the cells were fixed, washed twice with PBS, and then permeabilized with PBS containing 0.1% Triton X-100 for 5 min at 37°C. The cells were then stained with 50 *μ*L per well of DAPI 4′6-diamidino-2-phenylindole (Sigma, St. Louis, MO, USA) (1:2000 dilution) for 3 min of incubation at room temperature. Finally, the slides were washed with PBS and then assessed by using fluorescent microscopy.

### Statistical analysis

All data were analyzed by one-way ANOVA. Significant differences in means (*P *<* *0.05) were then compared by Duncan's test using the SPSS (SPSS Inc, Chicago, IL, USA) 19.0 software. All graphs were prepared by using Microsoft Office Excel.

## Results and Discussion

### Sampling, isolation, and morphological/biochemical characterization

The isolated single colonies in the MRS agar growth medium were hemispherically round with white or yellow color. By performing morphological and biochemical tests, the rod- or spherical-shaped gram-positive and catalase-negative single colonies that could belong to the *Lactobacillus* genus were isolated. A total of 125 bacteria were isolated from yogurt and colostrum. Of these, only 17 isolates belonged to *Lactobacillus* genus (Table1).

**Table 1:** Sequencing results for 17 representative isolates and survival rate of 17 representative isolates in simulated digestion condition and their capacity to adhere to Caco-2 cells

Isolates	Sequencing results	Dairy origen	Digestion survival (%)	Adhesion to Caco-2 (cfu/mL)
11C	*Lactobacillus delbrueckii*	Colostrum	0	0
17C	*Lactobacillus plantarum*	Colostrum	54	2.9 × 10^5^
13C	*Lactobacillus plantarum*	Colostrum	51	2.6 × 10^5^
12C	*Lactobacillus rhamnosus*	Colostrum	47	4.9 × 10^4^
5C	*Lactobacillus rhamnosus*	Colostrum	45	4.1 × 10^4^
1C	*Lactobacillus rhamnosus*	Colostrum	37	3.2 × 10^4^
15C	*Lactobacillus casei*	Colostrum	0	0
25C	*Lactobacillus casei*	Colostrum	0	0
36C	*Lactobacillus casei*	Colostrum	0	0
14C	*Lactobacillus paracasei*	Colostrum	0	0
32C	*Lactobacillus paracasei*	Colostrum	0	0
17M	*Lactobacillus casei*	Yogurt	0	0
20M	*Lactobacillus casei*	Yogurt	0	0
19M	*Lactobacillus paracasei*	Yogurt	0	0
13M	*Lactobacillus paracasei*	Yogurt	0	0
40M	*Lactobacillus plantarum*	Yogurt	32	2.8 × 10^4^
7M	*Lactobacillus rhamnosus*	Yogurt	48	2.6 × 10^4^

### Amplification and sequencing of 16S rRNA

The presence of *Lactobacillus* strains in the samples was confirmed by amplification of 1500 bp fragments of 16S rRNA gene with genus-specific primers. Among 17 strains, 11 were isolated from colostrum, whereas six were isolated from yogurt. According to results and FAO/WHO guidelines, identification of microorganisms by 16S rRNA patterns can be considered as a more suitable technique than other costly and time-consuming molecular techniques (Ben Amor et al. 2007). This technique has been effectively used for analyzing and isolating lactic and acetic acid bacteria from fermented dairy products (Pogačić et al. 2013; Tulini et al. 2013). The result confirmed that this method can be used as an accessible, low-cost, and suitable technique for isolating *Lactobacillus* strains from traditional dairy products.

The 1500 bp fragments of 16S rRNA gene were sequenced. By using BLAST software in NCBI site and comparing the results with deposited sequences in GenBank, we identified the isolates with 98–100% homology. The threshold value for taxonomical studies was approximately 97%. Although, 16S rRNA sequencing was a valid and accurate technique for phylogenetic clustering (Deng et al. 2008), the sequencing results should be compared with other molecular methods like GTG-PCR, ERIC-PCR, and ARDRA outcomes.

The strains belonged to five species of *Lactobacillus* genus (*L. delbrueckii, L. plantarum, L. rhamnosus*, *L. paracasei*, and *L. casei*). The results of our study and those of previous studies imply that the homology levels between some *Lactobacillus* strains reached more than 99% (Ritchie et al. 2010). Hence, this technique is valid for discrimination at the species level but is invalid and insufficient for discrimination at the strain levels. Sequencing results were comparable with those of other molecular methods, such as GTG-PCR, ERIC-PCR, and ARDRA. The biodiversity of *Lactobacillus* species in fermented dairy products is variable and region specific. In traditional Spanish cheese (Armada cheese), the predominant *Lactobacilli* are *L. casei* subsp*. casei* and *L. brevis* (Herreros et al. 2003). Meanwhile, in Greek goat cheese (Batzos cheese), *L. paracasei* and *L. sakei* are the dominant species (Psoni et al. 2003), whereas in Brazilian fresh cheese (Minas Frescal cheese) the predominant species is *L. acidophilus* (Lollo et al. 2012). It revealed that our results are quite different from those in terms of species and prevalence.

### Low pH and high concentrations of bile salts tolerance tests

Most probiotic bacteria are delivered through food. Therefore, these bacteria must survive for a minimum 90 min under low pH conditions or in the presence of secreted high concentrations of bile salts in the digestive system before colonization in the gastrointestinal tract and before displaying health-promoting effects. These defense mechanisms destroy the entering microorganisms, such that the assessment of resistance under harsh conditions is important (Sahadeva et al. 2011).

The in vitro low pH and high concentrations of bile salts tolerance experiments show the same cell viability results. The assessment of the harsh condition tolerance of probiotic bacteria can be performed by using an in vitro method with the same pH (pH 2.5) or concentration of oxgall [0.3% (w/v)] in the gastrointestinal tract (Ben Salah et al. 2012).

The survival rates for 17 isolated *Lactobacillus* strains after 3 h of incubation at pH 2.5 and the substance at 0.3% (w/v) high concentrations of bile salts are shown in Table2. All isolates displayed high growth at low pH and high concentrations of bile salts.

**Table 2:** The survival rates of strains after 3 h incubation at pH 2.5 and 0.3% bile salts

Isolates	Final counts (log CFU/mL) after incubation at		Final counts (log CFU/mL) after incubation at	
	0 h	3 h	0 h	3 h
11C	9.02 ± 0.02	5.86 ± 0.04	8.92 ± 0.03	6.96 ± 0.02
17C	8.96 ± 0.03	7.17 ± 0.03	9.05 ± 0.02	7.96 ± 0.03
13C	8.12 ± 0.02	6.17 ± 0.01	9.17 ± 0.05	7.98 ± 0.04
12C	8.47 ± 0.04	6.69 ± 0.02	8.99 ± 0.03	8.00 ± 0.03
5C	8.51 ± 0.05	6.47 ± 0.03	8.93 ± 0.02	8.04 ± 0.03
1C	8.77 ± 0.03	7.19 ± 0.03	9.07 ± 0.05	8.07 ± 0.02
15C	8.94 ± 0.01	5.54 ± 0.03	9.11 ± 0.05	6.65 ± 0.03
25C	9.07 ± 0.02	5.44 ± 0.05	8.88 ± 0.03	6.39 ± 0.05
36C	9.38 ± 0.04	6.00 ± 0.04	9.18 ± 0.04	6.98 ± 0.02
14C	8.99 ± 0.02	5.21 ± 0.02	9.09 ± 0.03	6.45 ± 0.03
32C	8.82 ± 0.04	5.29 ± 0.03	9.15 ± 0.03	6.50 ± 0.05
17M	8.69 ± 0.05	5.39 ± 0.05	8.93 ± 0.02	6.70 ± 0.02
20M	8.74 ± 0.05	5.68 ± 0.04	8.97 ± 0.03	6.55 ± 0.04
19M	8.78 ± 0.04	5.00 ± 0.04	8.77 ± 0.04	6.05 ± 0.03
13M	9.20 ± 0.03	5.80 ± 0.02	9.13 ± 0.04	6.57 ± 0.02
40M	9.15 ± 0.02	7.14 ± 0.03	9.17 ± 0.05	7.79 ± 0.02
7M	9.03 ± 0.04	7.22 ± 0.01	9.00 ± 0.02	8.19 ± 0.02

The isolates showed high survival rates of more than 57% of that of other strains under low pH conditions. The survival rates of the *L. plantarum* and *L. rhamnosus* strains (76–82%) were also significantly higher than those of the other strains (*L. delbrueckii, L. casei,* and *L. paracasei*), which ranged from 57% to 65%, at a 0.05 significance level. These results are in accordance with those of other studies, which showed that some *Lactobacillus* strains, such as *L. plantarum*, tolerate low pH conditions better than other strains (Abriouel et al. 2012).

The isolates displayed higher tolerance of high concentrations of bile salts conditions (≥69%). The high concentrations of bile salts tolerance capability was strain specific, similar to the case under acidic conditions. The effects of high concentrations of bile salts on bacterial cells can be distinguished from the acidic effects, but some combined results can be observed. Stress adaptation mechanisms caused the resistance to high concentrations of bile salts to be higher than that under acidic conditions (Sahadeva et al. 2011). The high tolerance capability of *Lactobacillus* strains under high concentrations of bile salts has been reported by other researchers (Pan et al. 2009).

Similar to those under acidic conditions, the survival rates for *L. plantarum* and *L. rhamnosus* strains (85–91%) were higher than those of the other strains (*L. delbrueckii, L. casei,* and *L. paracasei*) at a range of 69–78% at a 0.05 significance level.

### Survival in simulated in vitro digestion and adhesion assay to Caco-2 cells

One of the most desirable features of probiotics is their capability to remain alive in the gastrointestinal tract. A total of 17 isolated *Lactobacillus* were tested to evaluate the strains further through a simulated digestion test. Results showed that seven strains have considerable digestion survivability. The resistant strains were three *L. plantarum* and four *L. rhamnosus*. Five of these resistant strains were isolated from colostrum, whereas two were isolated from yogurt. The highest percentage of survivability was observed for *L. plantarum* 17C and 13C (both isolated from colostrum), with survivability values of 54% and 51%, respectively (Table2). Similar to our results, high survival rates were reported for three commercial probiotic strains including *L. casei* subsp. *shirota, L. casei* subsp. *immunitas,* and *L. acidophilus* subsp. *Johnsonii* in simulated digestion condition (Lo Curto et al. 2011).

These seven digestion-resistant LAB strains were examined for their capability to adhere to Caco-2 cells. The results showed that *L. plantarum* 17C and 13C were the most adherent strains, with adhesion values of 2.9 × 10^5^ (≈3 bacteria per Caco-2 cell) and 2.6 × 10^5 ^cfu/mL (≈2.5 bacteria per Caco-2 cell), respectively (Table1). These results are in accordance with those of other studies, which showed that some *L. plantarum* strains, such as *L. plantarum* L2, *L. plantarum* CH3 and CH41, could adhere to Caco-2 cells better than other *Lactobacilli* (Wang et al. 2009; Ramos et al. 2013).

A requirement for bacteria to be recognized as a probiotic is their capability to remain alive while passing through the upper digestive tract to reach the large intestine, where their useful actions are expected. To be colonized in the intestine, probiotic bacteria have to adhere to the intestinal mucosa to avoid being removed from the colon by peristalsis. Seventeen “dairy isolates” were examined for this test. Among these isolates, only seven strains survived exposure to the simulated digestion conditions of the stomach. The capability to adhere to Caco-2 cells was investigated for further analysis. We found that the strains *L. plantarum* 17C and 13C isolated from colostrum were the most resistant strains to digestion conditions and were the best strains in terms of adherence to Caco-2 cells. This result showed that these strains could be new potential probiotic candidates and that colostrum could be a suitable source of probiotics.

### Antimicrobial activity assay

The inhibitory characteristics of probiotics against pathogens can be considered as their most important health-promoting properties (Cizeikiene et al. 2013; Gerez et al. 2013). According to the diameter of the inhibition zone, the antipathogen activity was classified into strong (diameter ≥ 20 mm), moderate (20 mm > diameter > 10 mm), and weak (diameter ≤ 10 mm) (Lim 2010).

Among the 17 isolates, 15 isolates displayed moderate antimicrobial activities, whereas two isolates (*L. paracasei* 19M and *L. casei* 25C) did not exhibit any antagonistic activities. In contrast, gram-negative pathogens, such as *S. typhimurium* and *E. coli* (026), were more sensitive to extracted metabolites than gram-positive pathogens, such as *B. cereus* and *L. monocytogenes*. This finding can be linked to the thin cell walls of gram-negative pathogens and their susceptibility to acidic metabolites (Table3).

**Table 3:** Antimicrobial activity of isolates against the pathogenic bacteria

	Diameter of inhibition zone (mm)						
Bacteria	*Salmonella typhimurium*	*Staphylococcus aureus*	*Escherichia coli (026)*	*Bacillus cereus*	*Listeria monocytogenes*	*Klebsiella pneumoniae*	*Shigella flexneri*
*Lactobacillus delbrueckii* 11C	11.8 ± 0.4^a^	0.0 ± 0.0^b^	12.0 ± 0.6^a^	0.0 ± 0.0^b^	12.7 ± 1.2^a^	0.0 ± 0.0^b^	12.3 ± 0.3^a^
*Lactobacillus plantarum* 17C	12.3 ± 1.2^b^	11.7 ± 0.3^b^	12.3 ± 0.7^b^	10.0 ± 0.0^c^	13.7 ± 0.9^a^	12.0 ± 0.6^b^	12.0 ± 0.0^b^
*Lactobacillus plantarum* 13C	12.3 ± 0.7^bc^	12.0 ± 0.6^c^	12.0 ± 0.0^c^	11.7 ± 1.2^c^	14.0 ± 0.6^a^	13.3 ± 0.3^ab^	12.7 ± 0.0^bc^
*Lactobacillus plantarum* 40M	14.0 ± 1.0^b^	10.3 ± 0.3^d^	15.7 ± 0.3^a^	12.0 ± 0.0^c^	0.0 ± 0.0^e^	0.0 ± 0.0^e^	12.0 ± 1.0^c^
*Lactobacillus rhamnosus 12C*	15.3 ± 0.7^a^	0.0 ± 0.0^d^	13.0 ± 0.6^c^	0.0 ± 0.0^d^	14.0 ± 0.6^b^	0.0 ± 0.0^d^	0.0 ± 0.0^d^
*Lactobacillus rhamnosus 5C*	13.3 ± 0.3^c^	13.0 ± 0.0^c^	15.7 ± 0.3^b^	0.0 ± 0.0^d^	17.0 ± 0.0^a^	13.3 ± 1.2^c^	14.0 ± 0.6^c^
*Lactobacillus rhamnosus 1C*	16.7 ± 0.3^a^	13.0 ± 0.0^bc^	14.0 ± 1.0^b^	12.3 ± 1.2^c^	0.0 ± 0.0^d^	0.0 ± 0.0^d^	13.3 ± 0.3^bc^
*Lactobacillus rhamnosus 7M*	12.8 ± 0.8^a^	0.0 ± 0.0^b^	12.3 ± 1.2^a^	0.0 ± 0.0^b^	0.0 ± 0.0^b^	0.0 ± 0.0^b^	12.0 ± 0.6^a^
*Lactobacillus casei* 15C	0.0 ± 0.0^c^	0.0 ± 0.0^c^	0.0 ± 0.0^c^	0.0 ± 0.0^c^	0.0 ± 0.0^c^	13.3 ± 0.7^a^	11.0 ± 0.0^b^
*Lactobacillus casei* 25C	0.0 ± 0.0^a^	0.0 ± 0.0^a^	0.0 ± 0.0^a^	0.0 ± 0.0^a^	0.0 ± 0.0^a^	0.0 ± 0.0^a^	0.0 ± 0.0^a^
*Lactobacillus casei* 36C	0.0 ± 0.0^c^	0.0 ± 0.0^c^	13.7 ± 1.2^a^	0.0 ± 0.0^c^	0.0 ± 0.0^c^	12.7 ± 0.3^b^	0.0 ± 0.0^c^
*Lactobacillus casei* 17M	0.0 ± 0.0^b^	0.0 ± 0.0^b^	0.0 ± 0.0^b^	0.0 ± 0.0^b^	0.0 ± 0.0^b^	12.0 ± 0.0^a^	0.0 ± 0.0^b^
*Lactobacillus casei* 20M	15.0 ± 0.6^a^	0.0 ± 0.0^d^	11.0 ± 0.0^c^	0.0 ± 0.0^d^	0.0 ± 0.0^d^	12.3 ± 1.2^b^	12.0 ± 0.0^b^
*Lactobacillus paracasei* 14C	0.0 ± 0.0^c^	0.0 ± 0.0^c^	0.0 ± 0.0^c^	0.0 ± 0.0^c^	0.0 ± 0.0^c^	12.7 ± 1.2^a^	11.0 ± 0.0^b^
*Lactobacillus paracasei* 32C	12.0 ± 1.0^a^	0.0 ± 0.0^b^	0.0 ± 0.0^b^	0.0 ± 0.0^b^	0.0 ± 0.0^b^	0.0 ± 0.0^b^	0.0 ± 0.0^b^
*Lactobacillus paracasei* 19M	0.0 ± 0.0^a^	0.0 ± 0.0^a^	0.0 ± 0.0^a^	0.0 ± 0.0^a^	0.0 ± 0.0^a^	0.0 ± 0.0^a^	0.0 ± 0.0^a^
*Lactobacillus paracasei* 13M	0.0 ± 0.0^c^	13.7 ± 1.2^a^	0.0 ± 0.0^c^	0.0 ± 0.0^c^	0.0 ± 0.0^c^	13.0 ± 1.0^a^	11.3 ± 0.7^b^

*Salmonella typhimurium* (14028); *Staphylococcus aureus* (ATCC 25923); *Escherichia coli* (026) (native strain); *Bacillus cereus* (ATCC 11778); *Listeria monocytogenes* (PTCC 1163); *Klebsiella pneumoniae* (ATCC 10031); *Shigella flexneri* (PTCC 1234) values are mean ± standard error mean (SEM). All incubations were performed in triplicate. Within a row, values with different superscripts are significantly different at *P ≤ 0.05*.

The antimicrobial activities of *Lactobacillus* strains can be attributed to the secretion of different antipathogen substances, such as bacteriocins, biosurfactants, H_2_O_2_, and organic acids (hydrochloric, lactic, and acetic acids) (Giraffa et al. 2010).

Among the treated isolates, *L. plantarum* strains, particularly *L. plantarum* 17C and *L. plantarum* 13C, displayed good antagonistic activities, as reflected by the inhibition of all indicator pathogens. Similar to our results, high antagonistic activity was reported for *L. plantarum* strains isolated from fermented food and dairy products against different pathogens (Gupta and Srivastava 2014; Ryu et al. 2014).

### Antibiotic susceptibility assay

Antibiotic overuse has caused the antibiotic resistance genes to spread across regions and transfer onto probiotic societies, such that the sensitivity of probiotics to conventional antibiotics is a fundamental health-promoting characteristic which causes to avoid resistant genes flow to other pathogenic/nonpathogenic bacteria especially intestine microflora (Temmerman et al. 2003).

The antibiotic susceptibility of 17 isolates against the high consumption of clinically important antibiotics was assessed on the basis of the formation of inhibition zones (Table4). The results were expressed in terms of resistance, moderate susceptibility, or susceptibility by comparing with the interpretative zone diameters given by the performance standards for antimicrobial disk susceptibility tests (NCCLS, 2007).

**Table 4:** Antibiotic susceptibility of isolated bacteria against the high consumption antibiotics performed by disk diffusion assay

	Diameter of inhibition zone (mm)								
Isolates	C	TE	E	AM	GE	CC	SMZ	P	V
*Lactobacillus delbrueckii* 11C	28S	30S	28S	18S	13S	29S	22S	22S	28S
*Lactobacillus plantarum* 17C	25S	22S	22I	30S	28S	24S	24S	30S	0R
*Lactobacillus plantarum* 13C	30S	28S	21I	38S	12S	20S	22S	40S	0R
*Lactobacillus plantarum* 40M	22S	26S	26S	26S	30S	28S	0R	28S	0R
*Lactobacillus rhamnosus* 12C	14S	20S	25S	29S	16S	29S	0R	15I	26S
*Lactobacillus rhamnosus* 5C	14I	22S	0R	11R	14S	30S	22S	13I	0R
*Lactobacillus rhamnosus* 1C	13I	14I	26S	22S	0R	17I	0R	26S	13I
*Lactobacillus rhamnosus* 7M	36S	28S	0R	0R	22S	26S	20S	0R	0R
*Lactobacillus casei* 15C	28S	30S	26S	28S	30S	30S	0R	32S	0R
*Lactobacillus casei* 25C	20S	20S	20I	26S	18S	28S	15I	28S	0R
*Lactobacillus casei* 36C	22S	30S	0R	30S	22S	22S	30S	30S	0R
*Lactobacillus casei* 17M	0R	28S	28S	28S	0R	28S	26S	0R	0R
*Lactobacillus casei* 20M	28S	22S	25S	26S	24S	30S	32S	0R	0R
*Lactobacillus paracasei* 14C	22S	30S	0R	30S	18S	22S	20S	22S	20S
*Lactobacillus paracasei* 32C	30S	20S	30S	26S	30S	26S	0R	26S	26S
*Lactobacillus paracasei* 19M	22S	14I	26S	28S	20S	30S	0R	30S	22S
*Lactobacillus paracasei* 13M	20S	20S	28S	28S	22S	25S	26S	20S	18S

Erythromycin results based on R < 13 mm; I: 13–23 mm; S > 23 mm. Gentamycin results based on R ≤ 6 mm; I: 7–9 mm; S ≥ 10 mm. Vancomycin results based on R < 12 mm; I: 12–13 mm; S > 13 mm. Chloramphenicol, Tetracycline, Ampicillin, Clindamycin, Penicillin, and Sulfamethoxazol based on I: intermediate (zone diameter, 12.5–17.4 mm); R: resistant (zone diameter, ≤12.4 mm); S: susceptible (zone diameter, ≥17.5) (NCCLS, 2007). C, chloramphenicol (30 *μ*g); TE, tetracycline (30 *μ*g); E, erythromycin (15 *μ*g); AM, ampicillin (10 *μ*g); GE, gentamycin (10 *μ*g); CC, clindamycin (2 *μ*g); SMZ, sulfamethoxazol (15 *μ*g); P, penicillin (10 *μ*g); V, vancomycin (30 *μ*g).

Isolated *Lactobacillus* strains displayed high sensitivity to treated antibiotics. Meanwhile, resistance to tetracycline, cefixime, pefloxacin, neomycin, enoxacin, sulfamethoxazol, lincosamide, cloxacillin, penicillin G, streptomycin, gentamycin, erythromycin, and chloramphenicol was reported among *Lactobacillus* strains isolated from dairy products (Bernardeau et al. 2008).

All isolates were susceptible or intermediate to tetracycline and clindamycin. The high resistant strains to tetracycline among the probiotic bacteria were reported by other researchers (Chang et al. 2011). Despite limited reports on clindamycin resistance genes among probiotics (Casado Muñoz et al. 2014), these resistance genes can be easily transferred to pathogenic strains, such as *S. aureus*, and thus cause outbreaks of bacteremia (Dubey et al. 2013). The sensitivity to these antibiotics can probably be attributed to the limited use of antibiotics in the rural area of Iran (Kermanshah province), such that isolated probiotics can safely be consumed after antibiotic (tetracycline and clindamycin) therapy.

The maximum resistance was observed for vancomycin, more than half of the strains (10 strains) displayed high resistance. The strict homo-fermentative *Lactobacillus* species, such as *L. paracasei* strains, are sensitive to vancomycin, which agrees with our results. Other *Lactobacillus* strains, such as *L. casei* and *L. plantarum*, which are similar to our isolates, carry the vancomycin resistance genes (Bernardeau et al. 2008).

*L. delbrueckii* 11C and *L. paracasei* 13M displayed the best results and were sensitive or semisensitive to all antibiotics, whereas *L. rhamnosus* 7M and *L. casei* 17M, were the most resistant strains among isolates on the basis of their resistance to four antibiotics.

### Cell viability assay

The MTT method is commonly used to scrutinize the cytotoxicity and viability of living cells in a 96-well plate format. This evaluation was performed by assessing *L. plantarum* secretion metabolites on cancerous/normal human cell lines to determine their capability for growth inhibition using the MTT assay. Paclitaxel, a standard commercial drug for treating different cancers, was utilized as a positive control. The MTT assay is a colorimetric method based on the metabolic capability of cells to decrease the yellow tetrazolium salt 3-[4, 5-dimethylthiazol-2-yl]-2,5-diphenyltetrazolium bromide or MTT to a blue crystalline formazan product. This method is widely used to analyze the cytotoxicity and cell viability of living cells in a 96-well plate.

**Figure 1:**
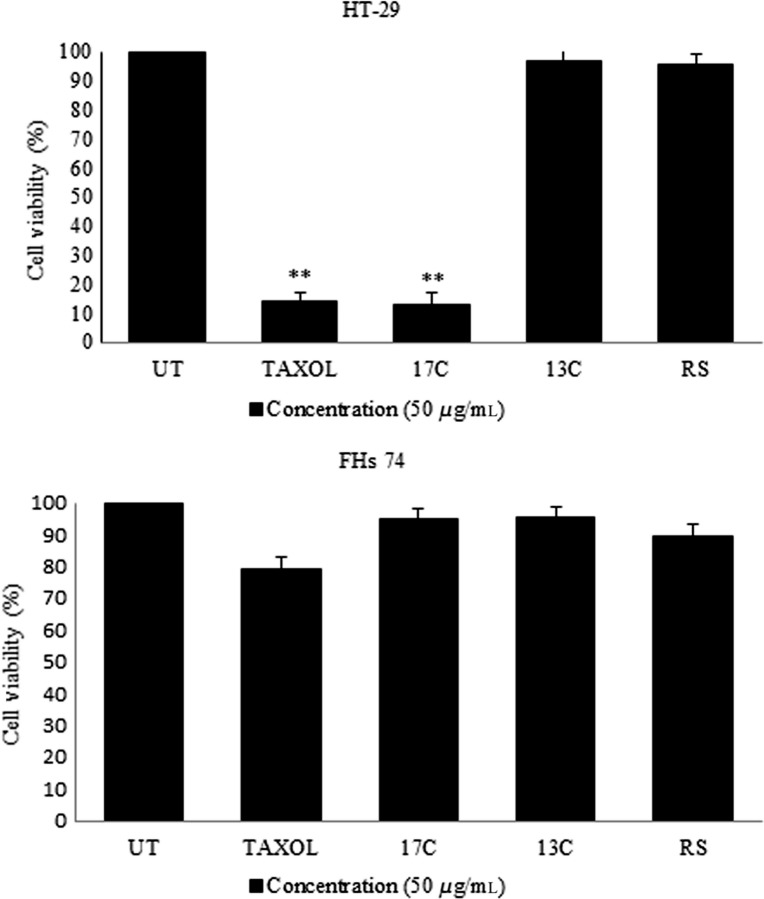
Effect of *Lactobacillus plantarum*17C and 13C supernatant on the viability of HT-29 cancer cells and FHs 74 normal cell by 50 *μ*g/mL concentration and 24 h incubation. Data are expressed as mean viability ratio ± SD. Asterisks denote statistically significant differences (**p ≤ 0.01; *p ≤ 0.05).All incubations were carried out in triplicate. UT means untreated and RS means reference strain *L. plantarum* ATCC 8014 to compare their effects.

Figure1A illustrates the cytotoxicity potential of *L. plantarum* 17C and 13C supernatant on HT-29 cancer cell. *Lactobacillus plantarum* ATCC 8014 was used as a reference strain for comparison. The viability of HT-29 carcinoma cells was inhibited by *L. plantarum* 17C secretions after 24 h of incubation, and the viable cells of HT-29 comprised 13% after 24 h of incubation. Our findings revealed that the antiproliferative effect of *L. plantarum* 17C secretion metabolites on HT-29 cancer cells had significant differences with that of the *L. plantarum* 13C and *L. plantarum* ATCC 8014 as control. We also used normal FHs 74 to determine the effect of this strain on normal cells (Fig.1B). No significant cytotoxic effects for these cells were observed, and approximately 94% of the normal cells grew well.

### Apoptosis detection by DAPI staining

Apoptosis is the primary means of programmed cell death and serves a significant function in regulating tissue development and homeostasis. Hence, the induction of apoptotic cell death is a favorable emerging scheme for the inhibition and treatment of cancer. Morphological changes offer the most direct criteria for distinguishing the apoptotic process. Thus, fluorescence microscopy was used to detect apoptosis on the basis of changes in cellular morphology, condensation and fragmentation of nuclei, as well as cell shrinkage and membrane blebbing.

**Figure 2:**
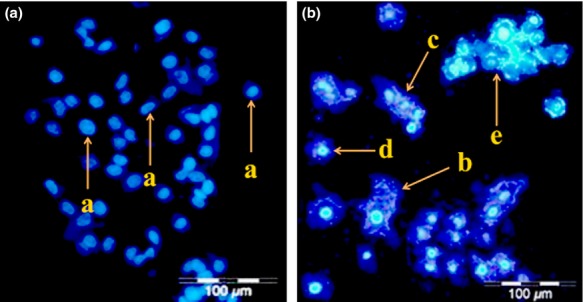
Detection of normal and apoptotic cells of HT-29 cancer cells without treating by *Lactobacillus plantarum* supernatant (A) and with treating by *L. plantarum* supernatant (B) after 24 h incubation. a: blue intact normal cell; b: membrane blebbing; c: nucleus fragmentation; d: cell shrinkage; e: apoptotic bodies.

To scrutinize the effect of *L. plantarum* 17C on HT-29 cell viability, HT-29 cells were exposed to the supernatant of the late stationary phase growth of *L. plantarum* 17C and analyzed by fluorescent microscopy (Olympus BX61). None of apoptotic-related signals were observed in the nontreated HT-29 cells (Fig.2A). Significant numbers of apoptotic cells were found in the cells after 24 h of incubation with 50 *μ*g/mL of *L. plantarum* 17C supernatant. The number of apoptotic cells with condensed and fragmented nuclei was significantly higher than that of blue intact normal cells. The treated HT-29 cells showed distinctive signs of apoptosis, including micronucleus formation, membrane blebbing, cell shrinkage, apoptotic bodies, and nuclear fragmentation, after 24 h of exposure (Fig.2B). Membrane blebbing and micronucleus formation were prominent apoptotic features of the HT-29 cells treated with *L. plantarum* 17C.

In conclusion, traditional yogurt and colostrum were preliminary screened because variable animal feeding and rare use of antibiotics can introduce novel and promising probiotic bacteria. Our findings indicated that *L. plantarum* 17C and *L. plantarum* 13C strains, which were isolated from colostrum, displayed a desirable tolerance to low pH and high concentrations of bile salts, favorable antipathogen activity, and acceptable antibiotic susceptibility. Thus, these two strains can be considered as potential probiotics. In addition, *L. plantarum* 17C showed significant antiproliferative effects on HT-29 human colon cancer cell line.
